# Early neonatal sepsis: prevalence, complications and outcomes in newborns with 35 weeks of gestational age or more

**DOI:** 10.1590/1984-0462/2022/40/2020388

**Published:** 2021-10-04

**Authors:** Juliana Fernandes de Camargo, Jamil Pedro de Siqueira Caldas, Sérgio Tadeu Martins Marba

**Affiliations:** a Centro de Atenção Integral à Saúde da Mulher, Campinas, SP, Brazil.; b Universidade Estadual de Campinas, Campinas, SP, Brazil.

**Keywords:** Infant, newborn, Neonatal sepsis, Cross-sectional studies, Shock, septic, Recém-nascido, Sepse neonatal, Estudos transversais, Choque séptico

## Abstract

**Objective:**

To analyze the incidence, complications, and hospital discharge status in newborns with ≥35 weeks of gestational age with early neonatal sepsis.

**Methods:**

This is a cross-sectional, retrospective study. Cases of early-onset sepsis registered from January 2016 to December 2019 in neonates with gestational age of 35 weeks or more were reviewed in a level III neonatal unit. The diagnoses were performed based on the criteria by the Brazilian Health Regulatory Agency (Anvisa), and the episodes were classified according to microbiological classification and site of infection. The following complications were evaluated: shock, coagulation disorders, and sequelae of the central nervous system. The conditions at hospital discharge were also assessed. The collected data were analyzed with the descriptive analysis.

**Results:**

In the period, early neonatal sepsis occurred in 46 newborns, corresponding to 1.8% of all newborns admitted to the neonatal unit, with a prevalence of 4/1,000 live births. Culture confirmed sepsis ocurred in three patients (0.3/1,000 live births), with the following agents: *S. pneumoniae, S. epidermidis* and *S. agalactiae*. As to site of infection, there were 35 cases of primary bloodstream infection, seven cases of pneumonia and four cases of meningitis. Most patients (78.3%) had at least one risk factor for sepsis, and all were symptomatic at admission. There were no deaths. Complications occurred in 28.2% of the cases, especially shock (10 cases – 21.7%).

**Conclusions:**

The prevalence of proven early neonatal sepsis was low. Despite the common occurrence of complications, there were no deaths.

## RESUMO

**Objetivo:**

Analisar a prevalência, as complicações e as condições de alta dos recém-nascidos ≥35 semanas com diagnóstico de sepse neonatal precoce.

**Métodos:**

Estudo transversal, com coleta retrospectiva de dados. Incluíram-se todos recém-nascidos com 35 semanas ou mais de idade gestacional, com diagnóstico de sepse precoce em um período de quatro anos (janeiro/2016 a dezembro/2019) em uma unidade neonatal nível III. Os diagnósticos realizaram-se segundo os critérios da Agência Nacional de Vigilância Sanitária e os episódios classificados segundo a confirmação microbiológica e o sítio de infecção. As complicações avaliadas foram: choque, distúrbio de coagulação e sequelas do sistema nervoso central. Também se avaliaram as condições de alta. Os dados coletados foram analisados utilizando estatística descritiva.

**Resultados:**

No período, 46 recém-nascidos apresentaram sepse precoce, correspondendo a 1,8% das internações e a uma prevalência de 4/1.000 nascidos vivos. Em três pacientes a sepse foi confirmada por culturas (0,3/1.000 nascidos vivos), respectivamente por *S. pneumoniae, S. epidermidis* e *S. agalactiae*. Quanto ao sítio de infecção, foram 35 casos de infecção primária da corrente sanguínea, 7 casos de pneumonia e 4 de meningite. A maior parte dos pacientes (78,3%) possuía pelo menos um fator de risco para sepse, e todos apresentaram-se sintomáticos. Não houve óbito. Complicações ocorreram em 28,2% dos casos, especialmente choque (10 casos – 21,7%).

**Conclusões:**

A prevalência de sepse neonatal precoce comprovada foi baixa. Apesar da ocorrência comum de complicações, não houve óbitos.

## INTRODUCTION

Early neonatal sepsis (ENS) affects a significant number of newborns (NB), and is associated with increasing morbidity and mortality rates in the first week of life. Around the world, it is estimated that the infection is responsible for 27.5% of neonatal deaths, reaching rates as high as 20/1,000 live births in countries with high neonatal mortality rates. It is known that the data are inaccurate, especially in developing countries, where many deaths occur in the household, without medical assistance.^1^ In Brazil, the records of neonatal sepsis as a cause of death add up to approximately 3,000 infants per year.^2^ The unfavorable outcome ranges according to gestational age, and prematurity is an additional risk factor.^3^

Nowadays, the incidence of ENS in infants born at term is of approximately 0.5/1,000 live births; this number doubles among late preterm newborns (PTNB), and is even more significant in PTNB<34 weeks, and NB with very low birth weight.^4^

ENS is defined as the infection that takes place from birth until 48-72 hours of life, and unless there is any strong evidence of any other mean of contamination, the infections diagnosed before 48 hours of life are considered of maternal origin.^5^ Even though positivity is low, the gold standard for diagnosis is the positive culture in the blood and/or cerebrospinal fluid (CSF).^6^

This diagnosis is often difficult, since the signs and symptoms are unspecific and can be confused with the conditions of birth itself, or of the adaptation to extrauterine life. Therefore, in several cases it is necessary to assume the diagnosis and institute the treatment based on clinical findings and unspecific laboratory examinations. However, the use of well-defined clinical and laboratory criteria can be the base to elaborate a more accurate diagnosis, thus preventing the unnecessary use of antimicrobials.

Even though they were established in 2013, the diagnostic criteria of infections related to neonatology health care were not used in studies to evaluate ENS.^7^ Previous local analyses have shown ENS prevalence rates that are similar to those in developed countries, diverging from the national reality, which motivated the elaboration of this study. Therefore, the objective of this study was to assess the recent prevalence of ENS, as well as to analyze the risk factors, complications and the outcomes in NB≥35 weeks in a tertiary neonatal unit (NU) in the past four years, according to the criteria from the Brazilian Health Regulatory Agency (Anvisa).^7^

## METHOD

This is a cross-sectional study, with retrospective data collection. We used a convenience sample and included all NB≥35 weeks diagnosed with ENS in a four-year period, hospitalized between January 2016 and December 2019. The neonatal unit is a high complexity one (level III), with 30 beds.

The patients diagnosed with ENS were located after a search in the internal computerized database, with posterior analysis of medical charts.

We assessed maternal, obstetric and neonatal variables to describe the population.

The maternal risk factors for ENS were: colonization by Group B streptococcus (GBS), premature labor (<37 weeks), rupture of ovular membranes ≥18 hours, maternal fever (intrapartum maternal temperature ≥38°C), maternal sepsis, physometry, current urinary infection without treatment, or treatment inferior to 48 hours, and clinical chorioamnionitis.^8^

The diagnosis and management of cases of ENS started with the presence of clinical symptoms suggestive of the disease in the assessed NBs, regardless of the presence of risk factors for ENS. Therefore, infectious screening was performed, which included blood sample collection for hemoculture, blood count and dosage of C-reactive protein (CRP). The blood samples were collected from two different locations, containing 1mL of blood each, injected in Bactalert bottles (BD BACTEC^®^ — Becton Dickinson, Sparks, MD, USA) and analyzed through automatic processing. According to the internal protocol of the service, children with risk factor for ENS, however asymptomatic, are not submitted to the laboratory screening test and remain under clinical observation at least for 48 hours, with objective serial physical examination and evaluation of vital signs.^8^

CRP dosage was serially measured by nephelometry, with a 24-hour interval between the two samples. CRP was considered normal when≤10 mg/L.^9^

CSF was collected from patients with clinical and laboratory conditions for the procedure, before the institution of antibiotic therapy, regardless of the positivity of the hemoculture and considering normality values according to Sarff.^10^

The leukogram was performed through the manual cell count on a blade, and the result was interpreted according to hours of life, assessing the absolute number of neutrophils and the ratio of immature neutrophils/total neutrophils^11^. Thrombocytopenia was defined with numbers below 100,000/mm^3^.

ENS was classified according to the site of infection and in case of confirmation by other culture. The ENS was confirmed in cases in which it was possible to identify the agent through hemoculture and/or CSF culture.

The diagnosis of clinical sepsis was defined by the presence of changes in laboratory tests (blood count and/or CRP) and by the negative result of hemocultures and CSF, besides the absence of evidence of infection in another site, and at least two of the following signs without any other known cause: thermal instability, apnea, bradycardia, food intolerance, respiratory distress, hyperglycemia, hemodynamic instability, hypoactivity or lethargy.^7^ In the diagnosis of complications, shock was established by the need for volemic expansion and/or use of vasoactive drugs.

According to the site of infection, ENS was classified as primary bloodstream infection (PBSI), pneumonia or meningitis, according to Anvisa.^7^ The diagnosis of meningitis was established for patients who presented with at least one of the following signs or symptoms, without any other known cause: thermal instability (axillary temperature higher than 37.5°C or lower than 36.0°C), apnea, bradycardia, bulging anterior fontanel, signs of involvement of cranial nerves, irritability, seizure; and at least one of the following: altered CSF examination with high leukocyte count and proteins, decreased glucose and/or bacterioscopy and/or positive culture.^7^

The radiological criterion was used to diagnose pneumonia. One or more of the chest X-rays was serial, with one of the following findings: persistent infiltrate, new or progressive (consolidation, cavitation or pneumatocele), associated with ventilatory complications and with at least three of the signs and symptoms (thermal instability, leukopenia or leukocytosis, changes in tracheal secretion, purulent secretion in aspiration, respiratory sounds or snoring and bradycardia).^7^

During diagnostic suspicion, the treatment was immediately instituted with penicillin G and aminoglycoside until the partial results of the cultures and the antibiogram. The patients remained hospitalized in the intensive care unit under surveillance and treatment for the infectious process, as well as the associated morbidities (hemodynamic, ventilatory and coagulation disorders).

The discharge status was classified as: death, inter-hospital transfer, or discharge to the household. The analyzed complications related to sepsis were: presence of shock, coagulation disorder (active bleeding associated with changes in the coagulation test) and sequelae in the central nervous system. It is worth to mention that all newborns are submitted to neurological examinations at hospital admission and discharge. During hospitalization, in case they show signs of worsening and neurological damage, these are investigated.

The collected data were analyzed by using descriptive statistics methods, and the categorical variables were expressed by absolute and relative frequencies (%). The continuous variables were shown by mean and standard deviation, or median and interquartile rate (IQR), according to the distribution of values, and the rates were shown by percentage or per one thousand live births.

For the statistical analysis of data, we used the Statistical Analysis System, version 9.2 (Cary, NC, USA).

The project was approved by the Research Ethics Committee of the institution, including an authorization to dismiss the informed consent form (authorization n. 89429418.8.0000.5404).

## RESULTS

We analyzed 46 patients with ENS; 41 (89.1%) were born in the hospital. In the assessed period, 10,228 children were born in the service, and the total number of hospitalizations was 2,556. Therefore, the sample corresponded to 1.8% of the hospitalizations in the unit. The prevalence of ENS was 4.0/1,000 live births, and of confirmed ENS, 0.31/1,000 live births. The neonatal demographic data are in Table 1.

**Table 1:** Neonatal demographic data of cases of early neonatal sepsis in the four-year period (2016 to 2019) (n=46).

	n (%)
Gestational age (weeks)*	38.0±1.7
Weight at birth (grams)*	3138±483
Weight at birth <2500g	5 (10,9)
Premature infants	10 (21.7)
Positive pressure ventilation at birth	11 (23.9)
Apgar<7 at 5 minutes	2 (4.3)
Male	24 (52.2)
Days in hospital**	10 (7.7–15.0)

Data is shown in absolute (N) and relative frequency (%) or

* in mean and standard deviation or

** in median and interquartile range

In the evaluation of maternal variables, mean age was 23.3 years, with prevalence of 54.3% of primiparous women, and 56.5% of vaginal birth. Most (43/46) were diagnosed with some morbidity during pregnancy, being: arterial hypertension (15.2%), diabetes mellitus (13.0%), urinary infection during pregnancy (37.0%) and other comorbidities (28.3%).

We found risk factors for sepsis in 36 patients (78.3%), who were assessed individually and described in Table 2; in 10 patients, there were no apparent risk factors. The research for GBS as prenatal screening was carried out in only 16 of the 46 NB mothers; 10 pregnant women had a positive result. Maternal intrapartum prophylactic antibiotics was performed in 23.9% (n=11) of the pregnant women, using crystalline penicillin (n=9), cefazolin (n=1) or clindamycin (n=1), with median of 7 hours (IQR 2-24) before birth. Among the women who did not receive prophylaxis, the justifications were: birth in another service (n=5), fever very close to the birth (n=6), diagnosis of physometry only at the time of birth (n=6), or no indication for the service protocol (n=12).^8^

**Table 2:** Risk factors in patients diagnosed with early neonatal sepsis during the four-year period (2016 to 2019).

	n
Rupture of membranes ≥18h	2 (4.3)
Colonization by GBS	6 (13.0)
Corioamnionitis	1 (2.2)
Maternal fever	6 (13.0)
Physometry	5 (10.9)
Current urinary infection	3 (6.5)
Maternal sepsis	3 (6.5)
Premature delivery	4 (8.7)
More than one risk factor	6 (13.0)

Values expressed in absolute (N) and relative frequency (%); GBS: Group B streptococcus — of the 10 positive pregnant women, 4 had more than one risk factor.

The diagnoses were analyzed in two manners: first, according to the microbiological confirmation, only three patients (6.5%) had a confirmed diagnosis of sepsis through hemocultures and/or CSF culture. The isolated agents in hemocultures were: *S. pneumoniae, S. epidermidis* and *S. agalactiae. S. pneumoniae* was also isolated in the CSF culture of the corresponding patient. The patient diagnosed with ENS due to *S. epidermidis* did not present evident maternal risk factors, and there was no amniotic puncture or other invasive procedures in the pregnant woman. Then, according to the site of infection, 35 cases of PBSI were identified (76.1%), 7 pneumonias (15.2%) and 4 cases of meningitis (8.7%).

All patients were symptomatic at some point of the evolution, being 71.7% of the patients symptomatic at birth; 17.4%, in the first 24 hours of life; and 6.5%, between 24 and 48 hours of life. It is worth to mention that respiratory distress was the most prevalent clinical sign, present in 89.1% of the patients with ENS and in all cases diagnosed with pneumonia. The main clinical signs are described in Table 3.

**Table 3:** Clinical presentation of patients with early neonatal sepsis during the four-year period (2016 to 2019).

	PBSI (n=35)	Pneumonia (n=7)	Meningitis (n=4)	n (%)
Respiratory distress	31	7	3	41 (89.1)
Thermal instability	10	1	3	14 (30.4)
Hypoactivity	6	3	1	10 (21.7)
Hypotension	6	4	0	10 (21.7)
Paleness	4	2	0	6 (13.0)
Food intolerance	4	1	0	5 (10.9)
Oliguria	3	1	0	4 (8.6)
Apnea	2	1	0	3 (6.5)
Irritability	2	0	0	2 (4.3)
Tachycardia	2	0	1	3 (6.5)
Tremor	0	0	2	2 (4.3)
Hypotonia	2	0	1	3 (6.5)
Seizure	1	0	1	2 (4.3)

Values expressed in absolute (N) and relative frequency (%); PBSI: primary bloodstream infection.

For the three patients with proven ENS, the symptoms were: hyperthermia (n=2), respiratory distress (n=2) and seizure (n=1).

All patients diagnosed with pneumonia presented with respiratory distress, lung auscultation, besides the radiographic changes documented in two different moments. Among them, 85.7% (n=6) needed ventilatory support by mechanic ventilation, and 57.1% (n=4) needed hemodynamic support with vasoactive drugs, given the state of shock. All patients with pneumonia presented with a systemic manifestation of the infectious process, being then clustered as early neonatal infection.

Seizure was considered as secondary to meningitis, proven in only one of the cases. The other patient presented normal CSF analysis, and the seizure was associated with the diagnosis of hypoxic ischemic encephalopathy.

The most frequently altered laboratory parameter was the number of total neutrophils (n=33); 17.3% of the NB presented with neutropenia, and 54.3%, with neutrophilia. The neutrophil index (ratio of immature neutrophil/total neutrophil count) was altered in 19 patients (41.3%). The presence of thrombocytopenia was described in only one case.

The CRP dosage was abnormal in the first collection in 54.3% of the samples. Of the 21 doses that were normal at first, 66% were altered in the second sample.

When it was possible to collect CSF (n=26), four patients presented changes in cytology and biochemistry, and the CSF culture was positive in only one patient (*S. pneumoniae*). The lumbar puncture was not performed in patients with respiratory, hemodynamic instability and/or coagulation disorders.

Complications were observed in 13 patients: hemorrhagic disorders (1), neurological sequelae after meningitis (2) and shock (10). The patients with shock received, in variable combinations and doses, dopamine (9), dobutamine (6), and adrenaline/noradrenaline (5). Four patients presented with refractory shock, requiring two or more drugs and association of hydrocortisone to the treatment.

The neurological changes that are secondary to the infectious process were observed in two patients: ventriculitis and ischemic stroke. Ventriculitis was shown in the brain ultrasound, corroborated by cranial tomography and characterized by a slight enhancement of the ependymal surface coating the lateral ventricles, and isoattenuating areas inside the ventricles (debris), besides the presence of dilation of the ventricular system, periventricular hypodensity, compatible with transudation in the CSF, and discreet enhancement in the contrast medium of the leptomeninge, especially in the bilateral frontoparietal high convexity. The diagnosis of post-infection ischemic stroke was carried out by nuclear magnetic resonance, with presence of areas of restriction for the diffusion of water molecules involving the cortex of the left superior and middle frontal gyrus, suggestive of ischemic injuries, probably secondary to vasculitis and to the complications of the infectious process.

Antimicrobial therapy was immediately instituted after the diagnosis of ENS, and the duration of treatment was determined according to the site of infection. The used antibiotics were: crystalline penicillin G or ampicillin (n=45), amikacin (n=38), cefotaxime (n=6), cefepime (n=1) and vancomycin (n=1). The median time of treatment was seven days (IQR 7-10), with variation of 7-21 days. The duration of treatment for all cases of clinical sepsis was seven days (100%), and for the cases of pneumonia, ten days (100%). Among the cases of meningitis, time of treatment ranged between 10 (n=2), 14 (n=1) and 21 days (n=1).

There were no deaths during hospitalization. Regarding the discharge status, 43 patients were discharged from the hospital, and 3 were transferred to the hospital of birth.

## DISCUSSION

The prevalence of ENS found in this study is similar to the numbers found in a recent North-American study, in which the rate of ENS ranged from 1 to 4/1,000 live births, depending on the region of the USA and the time of assessment.^12^ Regarding the prevalence of proven ENS, the observed value of 0.3/1,000 live births was lower than the one found in an extensive epidemiological study from the United Kingdom (0.71/1,000 live births)^13^, as well as the findings in the Brazilian study by Freitas et al. (1.7/1,000 live births)^14^. This difference can be explained by the inclusion of infants born at term or later PTNB in this study, whereas the other analyses described the global prevalence of ENS, without distinction between gestational ages, once the prevalence is inversely proportional to this factor. The choice of the gestational age ≥35 weeks was based on the national pattern of classification of candidates to rooming-in.

In the national scenario, there are few studies that determine the prevalence of ENS. A study from the state of Amazonas described an incidence of ENS of 53/1,000 live births, with a prevalent sample of NB>34 weeks of gestational age (95% of the cases)^15^. With a similar result, Goulart et al. described a rate of ENS of 50.3/1,000 live births, being 54% from the sample of NB>34 weeks, as well as that found by Benincasa et al. (46/1,000 live births)^16^ ^,^ ^17^. Therefore, the high rates of ENS that were previously described raise the question of the importance of well-defined diagnostic criteria, in order to determine the real rate of the disease incidence. Besides, it is important to establish treatments only for truly ill NB, and to promote the rational use of antimicrobials.

In developed countries, the number of patients with ENS has decreased throughout the past decades, and much of that is owed to the institution of antibiotic prophylaxis at the time of birth. This association was demonstrated in the multicenter study by Schrag et al., who showed a 65% reduction in the diagnosis of ENS from 1993 to 1998, after the elaboration of antibiotic prophylaxis guidelines for GBS.^18^

Only one case of confirmed ENS by GBS was described in the study. This micro-organism is the main agent of ENS in infants born at term.^12^ ^,^ ^13^ However, the prevalence of the disease has decreased with peripartum antibiotic prophylaxis. A Brazilian study, conducted in Brasília, showed prevalence of 1.7/1,000 live births.^14^ In the hospitalization unit of the study, this prophylaxis routine has already been instituted for over a decade, and this could be a factor to justify the occurrence of only one case of ENS by GBS. A local study in the early 2000s showed an incidence of 10.8/1,000 live births,^19^ however, involving mainly PTNB.

The positive cultures for *S. epidermidis* and *S. pneumoniae* differ from the ENS agents that are often described in the literature.^20^ Neonatal infection by pneumococcus is unusual.^13^ ^,^ ^21^ ^,^ ^22^ Besides, ENS caused by this agent is described as severe, with high mortality rates.^21^ As described in the literature, the patient developed the severe form, with motor sequelae and signs of cerebral ischemia in the nuclear magnetic resonance.

The infection by coagulase-negative staphylococci is usually related to prematurity and low weight, besides being a frequent cause of late sepsis.^23^ The agent is rarely described in ENS.^13^ ^,^ ^24^ Because they are commensal bacteria of the human skin, they are considered as contaminants of hemoculture samples when present in a single sample, or with late growth. In the study in question, the isolated *S. epidermidis* was considered as an ENS agent for being present in two samples of different hemocultures, due to time of early positivity (<24 hours) of the cultures, and for having been associated to a compatible clinical process and unspecific altered laboratory examinations.

The positivity of the cultures was low, similar to what the literature describes. According to Vergano et al., the infections with negative cultures are the most frequent ones, and can be confirmed in less than 1% of the cases.^25^ Other studies found a positivity rate of 2.3^17^, 3.0^26^ and 4.6%.^27^ The optimization of the laboratory diagnosis requires fast tests, with better sensitivity and high specificity, which still do not exist.^28^

A study highlight was the rigorous interpretation of the infectious screening tests, especially the blood count and the CRP, which were analyzed according to appropriate values for neonatal age, and not with standard values for older children, nor with established count numbers and neutrophil indexes, considering that the observed changes can reflect the conditions of the mother, the delivery and other diseases from the neonatal period.^11^

It is known that the evaluation of the blood count and CRP have a better negative than positive predictive value, and studies suggest that serial CRP can help to diagnose ENS with negative cultures.^29^

Given the low specificity of the current infectious screening tests, and the low positivity of cultures, which leads to the diagnosis of clinical sepsis in several cases, new studies that evaluate the study of genetic material and identification of bacterial particles have been promising. A Brazilian study that used universal primer (q-CRP) showed positivity in all patients diagnosed with clinical sepsis, and in 97% of the studied patients, the DNA of one or more among the seven analyzed bacteria was found.^26^ A remark for these tests is that the positive result can represent only one contamination, and, if performed in patients without any clinical indication, it would possibly lead to excessive treatment indications.

It is worth to mention that all patients diagnosed with ENS presented some type of symptom during hospitalization, and most of them were symptomatic at birth. The presence of symptoms in the first hours of life is considered as a warning sign for early sepsis. Besides, there is no indication for laboratory investigation and treatment at the absence of clinical signs of infection.^5^ The service follows the orientation for assessment based on risk stratification and the presence of symptoms since the 1990s, and is now corroborated by the American Academy of Pediatrics.^3^

The risk factors were absent in 20% of the cases, whereas the presence of symptoms was common to all of the assessed patients. The association of symptoms with risk stratification should be a path for the indication of treatment; however, the lack of risk factors should not be used as an exclusion criterion of ENS, especially in patients who are ill.

The investigation and the treatment of asymptomatic patients, based only on the presence of risk factors, expose many patients to the unnecessary use of antibiotics, besides separating the binomial, the impact on breastfeeding, the occupation of hospital beds and additional financial resources.^20^

Another strong aspect of the study is that the definition of suspected or confirmed cases of ENS was based on criteria that were determined by a national surveillance regulatory body, making the diagnosis more accurate, which enables the rational use of antimicrobials. The document with the national criteria to diagnose infections related to care and health is easy to access and has been available for two decades. The application of this method in other services could improve diagnosis and care in suspected and/or risk cases for ENS, without spending additional costs to the health service.

The clinical presentation should be valued, considering that symptoms are unspecific and can reflect ENS, as well as other diseases that can occur in the neonatal period of non-infectious causes.^20^ Besides, these conditions can often coexist with the infectious process, so the follow-up and evolution of cases are important and clarifying.

In a North-American study, which assessed all NB diagnosed with ENS born at the location, in a four-year period (2006 to 2009), the general mortality associated with ENS was 16%. However, in the subgroup of NB>37 weeks, the occurrence of death was low, approximately 3%.^12^ The absence of deaths in this study may reflect that proper care was provided to the children. However, this analysis was affected by the limited sample size. Another study limitation was the absence of assessment of suspected cases of ENS, which were ruled out afterwards due to the favorable clinical evolution and negative cultures 48 hours after collection. Besides, external validity is compromised since this study was carried out in a single center, of tertiary care, and with proper conditions for care and treatment of severe cases, which is not always the reality in national hospitals.

In conclusion, the prevalence of ENS in NB with 35 weeks or more of gestational age was similar to the data from developed countries, and lower when compared to the few descriptions in the national scenario. Despite being a disease that is known for its high morbidity and mortality rate, the complications we found were according to the expected for such a condition, and there was no death in the four assessed years.
